# Pretrained transformer models for predicting the withdrawal of drugs from the market

**DOI:** 10.1093/bioinformatics/btad519

**Published:** 2023-08-23

**Authors:** Eyal Mazuz, Guy Shtar, Nir Kutsky, Lior Rokach, Bracha Shapira

**Affiliations:** Department of Software and Information Systems Engineering, Ben-Gurion University of the Negev, P.O.B. 653, Beer-Sheva, 8410501, Israel; Department of Software and Information Systems Engineering, Ben-Gurion University of the Negev, P.O.B. 653, Beer-Sheva, 8410501, Israel; Department of Software and Information Systems Engineering, Ben-Gurion University of the Negev, P.O.B. 653, Beer-Sheva, 8410501, Israel; Department of Software and Information Systems Engineering, Ben-Gurion University of the Negev, P.O.B. 653, Beer-Sheva, 8410501, Israel; Department of Software and Information Systems Engineering, Ben-Gurion University of the Negev, P.O.B. 653, Beer-Sheva, 8410501, Israel

## Abstract

**Motivation:**

The process of drug discovery is notoriously complex, costing an average of 2.6 billion dollars and taking ∼13 years to bring a new drug to the market. The success rate for new drugs is alarmingly low (around 0.0001%), and severe adverse drug reactions (ADRs) frequently occur, some of which may even result in death. Early identification of potential ADRs is critical to improve the efficiency and safety of the drug development process.

**Results:**

In this study, we employed pretrained large language models (LLMs) to predict the likelihood of a drug being withdrawn from the market due to safety concerns. Our method achieved an area under the curve (AUC) of over 0.75 through cross-database validation, outperforming classical machine learning models and graph-based models. Notably, our pretrained LLMs successfully identified over 50% drugs that were subsequently withdrawn, when predictions were made on a subset of drugs with inconsistent labeling between the training and test sets.

**Availability and implementation:**

The code and datasets are available at https://github.com/eyalmazuz/DrugWithdrawn.

## 1 Introduction

Today, the cost of developing a single drug exceeds 2.6 billion dollars. But the investment required is not solely financial, as it takes an average of around 13 years to bring a drug to market (DiMasi *et al.* 2016). Identifying a new drug requires over 100 000 candidate compounds, as well as *in vitro*, *in vivo*, and three-phase clinical trials on thousands of subjects. Approximately 1 out of 10 compounds succeed in clinical trials, which means the success rate is around 0.0001% (Kapetanovic 2008).

In some cases, a drug that has shown effectiveness in clinical trials and has been approved by the US Food and Drug Administration (FDA) will have positive results in some patients, but for others, the drug may result in severe unwanted side effects, and in the worst case, even death (Lazarou *et al.* 1998, Wysowski and Swartz 2005, Ma and Lu 2011). Severe adverse drug reactions (ADRs) occur in 6.2%–6.7% of hospitalized patients, with over two million cases in the general population occurring annually in the USA alone. These ADRs result in 100 000 deaths per year in the US (Lazarou *et al.* 1998, Wilkinson 2005). Therefore, many drugs causing unexpectedly severe ADRs are eventually withdrawn from the market, with considerable impact on the producer, including a loss of revenue and reputation damage. Of the 548 drugs approved by the FDA between the years 1975 and 1999, 56 (10.2%) received a boxed warning or were eventually withdrawn from the market, and 20 (3.8%) of the 528 drugs approved between 1990 and 2009 in Canada were withdrawn for safety reasons (Lasser *et al.* 2002, Ninan and Wertheimer 2012).

Identifying safety issues in drugs is a challenging task. Each clinical trial phase can include up to a few hundred patients. In the early stages of drug discovery, computational approaches are used to identify potential drug molecules, reducing costs and time. Virtual screening (VS) is a powerful computational approach in which comprehensive libraries of small molecules are screened for new hits with desired properties that can be further investigated (Shoichet 2004).

Among the various VS approaches, quantitative structure-activity relationship (QSAR) analysis is a ligand-based drug design method. It attempts to find a statistically significant correlation between the chemical structure and biological and toxicological properties based on regression and classification techniques (Cherkasov *et al.* 2014). Despite their efficacy in identifying potential drug candidates, these techniques often overlook potential safety issues of the drugs. Furthermore, it’s important to note that QSAR models find applications not only in the pharmaceutical field but also in industrial chemistry, where they assess ecotoxicity, and in the field of materials science (Leszczynski 2006, Puzyn *et al.* 2010).

ADR prediction has received much attention in recent years. Prior studies utilized diverse information such as biological pathways (Scheiber *et al.* 2009), chemical-protein interactions (LaBute *et al.* 2014), and post-market surveillance data (Tatonetti *et al.* 2012) to predict ADRs. Despite their use, most of these types of data are based on experimental results or post-market reports, which require significant time and expense to gather and are unavailable in the early stage of the drug lifecycle. (Whitebread *et al.* 2005, Shtar 2021). But the reality is, to predict ADRs for a drug candidate at an early stage of drug development, predictions need to be made using just the chemical structure (Pauwels *et al.* 2011, Liu *et al.* 2014).

ADR events can lead to the withdrawal of a drug from the market, but that is not the only reason for withdrawing a drug; post-market drug withdrawals may be caused by a variety of factors, ranging from safety concerns such as reported deaths, to a wide range of non-safety concerns including the product’s inefficacy and a variety of manufacturing, regulatory, or business issues (Magro *et al.* 2012). Onakpoya *et al.* (2016) reported that the average time it takes for a drug to be withdrawn from the market has decreased; however, the method of identifying these drugs after a serious ADR has not improved in the past 60 years. In addition, the methodology used to identify previously unknown risks, as well as the delay between the introduction of a drug and its withdrawal for safety reasons, remain sources of concern. No gold standard for predicting drug withdrawal has been described in the literature.

A few *in silico* methods for predicting drug withdrawal have been proposed. Onay *et al.* (2017), who focused on drugs associated with nervous system diseases, were the first to distinguish between approved drugs and withdrawn ones. Their study combined Their study integrated the ToxPrint chemotype library (Yang *et al.* 2015), which was used to profile chemical datasets, with molecular descriptors derived from the compound structure, delineating a toxicity space.

For the classification task, support vector machines (SVMs) and ensemble methods were applied, and the authors concluded that several drug properties may be useful in discovering new medicines and that these properties can be used to filter out ADRs.

These models, however, are likely to fail in real-world applications due to the small datasets used to train them. In a large-scale benchmark study, Yang *et al.* (2019) demonstrated that a graph convolutional model constructed from a molecular graph has the potential to predict any molecular property. This predictive ability could be particularly valuable in the context of drug development. For instance, even with substantial pre-market evaluation efforts, including long and costly clinical trials, post-market drug withdrawals are generally not preventable by any existing means (Shah 2006, Daggumalli and Martin 2012). This highlights the potential benefit of utilizing advanced prediction models, such as the graph convolutional model, which could contribute to earlier and more accurate identification of drugs likely to be withdrawn, and hence, facilitate a more effective drug development process.

When using pretrained models on large datasets and adapting them for specific tasks, large language models (LLMs) such as transformers have demonstrated promising generalization abilities (Devlin *et al.* 2018). In recent years, They have become the gold standard in natural language processing (Vaswani *et al.* 2017); transformers have also become the model of choice in other domains such as computer vision (CV) as well as drug discovery (Bagal *et al.* 2021, Liu *et al.* 2021). For drug discovery, encoder-based transformers have been used; in this case, the model is pretrained on large datasets of molecules, and then it is optimized for property prediction (Chithrananda *et al.* 2020, Ahmad *et al.* 2022, Lu and Zhang 2022).

In this article, we propose using pretrained LLMs for predicting the probability of a drug being withdrawn from the market, using only the drug SMILES string sequence (Weininger 1988). Using several pretrained LLMs, each trained on a different task, we show that the representation learned in the pretraining phase can improve the prediction of drug withdrawal from the market. To demonstrate our models’ effectiveness in predicting withdrawn drugs and its robustness in real-world cases, we created a large database of drugs that were withdrawn from the market, performed a cross-database evaluation on three databases, and compared our models’ predictive performance with various types of baselines and models. Our results show that the proposed LLMs outperform other models in terms of the area under the curve (AUC) and Precision–Recall AUC (PR-AUC) scores.

The key contributions of our work are as follows.

- Demonstrate the potential of pretrained LLMs for drug withdrawal prediction.
- Create a comprehensive database of drugs withdrawn worldwide, consisting of drugs obtained from various public resources, the database is publicly available. The database is publicly available.

## 2 Materials and methods

### 2.1 Data collection

To create our data collection, we relied on several publicly available drug resources. We used DrugBank (Wishart *et al.* 2018) and ChEMBL (Mendez *et al.* 2019), two well-known bioinformatics databases that contain annotations of pharmaceutical market withdrawals; these databases also provide detailed drug information, including drugs’ chemical structure, pharmacological mechanism, targets, metabolic enzymes, carriers, and transporters. Additionally, we used Inxight Drugs’ NCATS (2020), a drug resource developed by the US National Center for Advancing Translational Sciences (NCATS), which contains the most comprehensive subset of substances and related biological mechanisms. We considered drugs marketed in the USA that were tagged as: marketed, withdrawn, or discontinued. We also obtained relevant drugs from WITHDRAWN, a resource for withdrawn and discontinued drugs, which was presented by Siramshetty *et al.* (2016) based on their systematic review of withdrawn drugs. A total of 626 drugs are included in the database, as well as their structures and key physicochemical characteristics.

In our study, we adopted the definition of DrugBank and define a drug as “withdrawn” if it was removed, banned, or rejected for marketing by at least one country for any reason. The label “not withdrawn” is applied to all of the drugs annotated in the DrugBank database as approved for marketing and not withdrawn by any of the stated resources (a total of 2346).

### 2.2 Dataset preprocessing

The Simplified Molecular-Input Line-Entry System (SMILES) (Weininger 1988), it is a short string describing the composition, connectivity, and charge of atoms in a compound (e.g. the SMILES for vanillin is: COc1cc(C = O)ccc1O). The SMILES is the most fundamental data possessed at the beginning of the drug discovery process.

**Figure 1.**
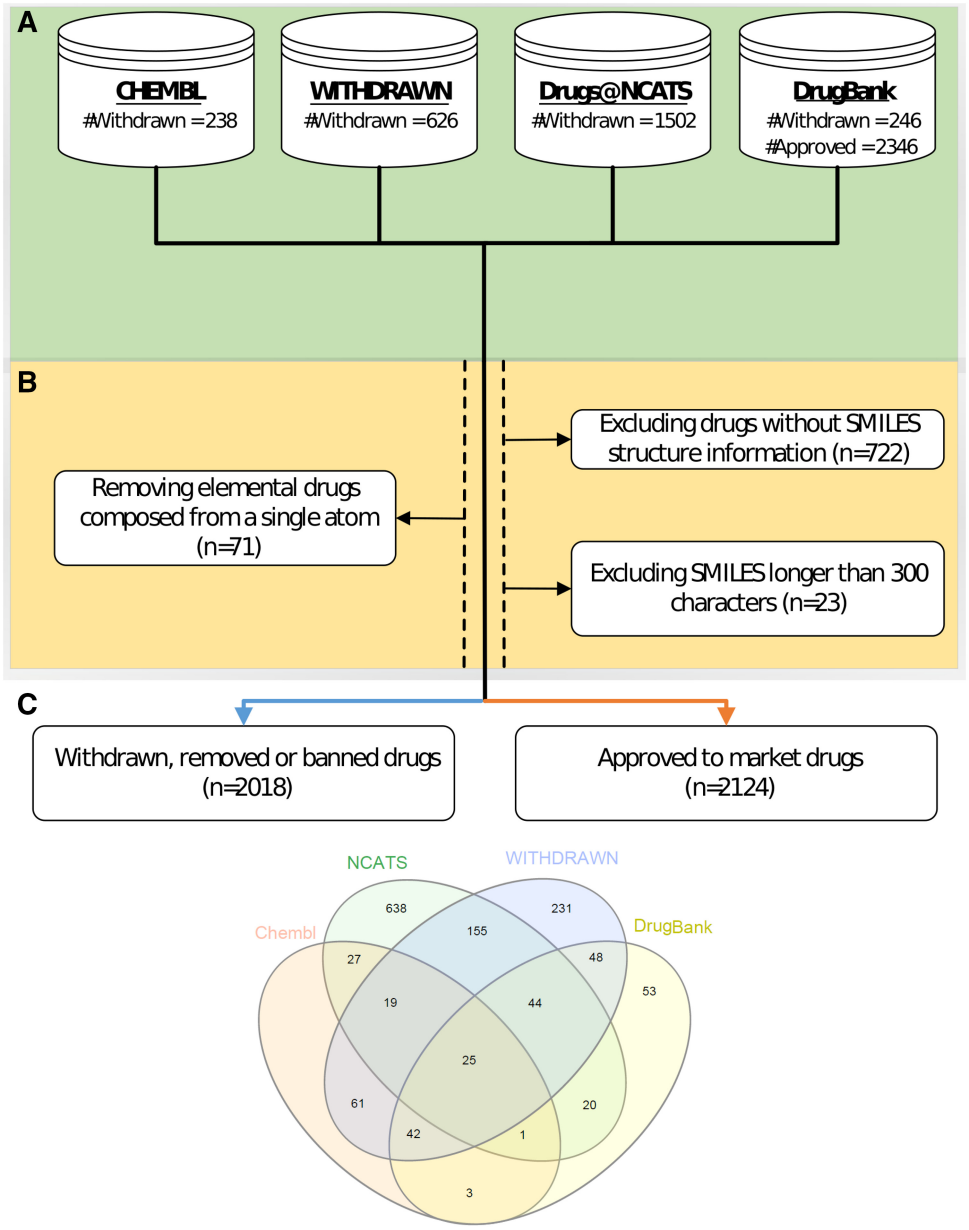
Data collection and processing pipeline. (A) The public sources we used for drug collection and the number of drugs in each (withdrawn/approved). (B) Exclusion of drugs as part of the preprocessing stage. (C) Total drugs, along with a Venn diagram of the number of withdrawn drugs from each of our sources.

**Standardization:** The drugs in each database were standardized by applying the following workflow: first, we removed duplicates and kept those drugs for which the SMILES structure was available. Next, we removed drugs that consist of a single type of atom (e.g. oxygen, iron) and kept only drugs of a more complex form (compounds). In addition, we removed SMILES longer than 300 characters, since they are outliers in our dataset and only add to the model complexity. Finally, we were left with a total of 4142 drugs, 2018 of which were labeled as “withdrawn” and 2124 drugs that were labeled as “not withdrawn” (see Fig. 1).

### 2.3 Large language models

In this section, we describe the transformer architecture, which is composed of a stack of transformer blocks, each learning a unique representation via the self-attention mechanism; we describe the formulation of the self-attention mechanism and the components that form the transformer block. Then, we present transformer-based various architectures that are built on this formulation.

**Embedding**: We first transform each token in our SMILES input to an embedding vector, using an embedding layer with a shape *W^NxD^* where *N* is the number of tokens, and *D* is the embedding dimensions.

**Attention:** The attention mechanism receives a set of keys, queries, and values (*q*, *k*, *v*) as inputs and applies a dot product operation between the queries and the keys to create compatibility weights. The output is then computed as a weighted sum of the query-key weights and the values. The attention mechanism is formulated as follows:


(1) $$\mathrm{Attention}(Q,K,V)=\mathrm{softmax}(\frac{QK^{T}}{\sqrt{d_{k}}})V.$$

Where *Q*, *K*, and *V* are matrices of stacked queries, keys, and values, respectively. Here, *q*, *k*, and *v* represent the individual elements in the matrices, each with a size of *d^k^*, where $k\in d^{k}$ and $v\in d^{k}$. These values are equal to $d_{\text{model}}$, which denotes the size of the linear layer in the model. This simultaneous computation enables us to compute the attention function for all inputs.

In order to learn different representations, we use multi-head attention (Vaswani *et al.* 2017), which allows us to incorporate information for different positions at the same time: where $W_{i}^{Q},W_{i}^{K},W_{i}^{V}$ are the projection matrices of head *i*. We can then define a transformer attention block as follows: where $x_{l-1}$ is the input from the previous block, MLP is a multi-layer feed-forward network, and MHA is the MultiHeadAttention. These blocks can now be stacked to create the transformer’s encoder architecture.


(2) $$\mathrm{MultiHead}(Q,K,V)=\mathrm{Concat}(\mathrm{hea}d_{1},\ldots ,\mathrm{hea}d_{n})W^{o},$$

(3) $$\mathrm{hea}d_{i}=\mathrm{Attention}(QW_{i}^{Q},KW_{i}^{K},VW_{i}^{V}),$$

(4) $$\begin{array}{c} z_{l}=x_{l-1}+\mathrm{MHA}(\mathrm{LayerNorm}(x_{l-1})),\\ x_{l}=z_{l}+\mathrm{MLP}(\mathrm{LayerNorm}(z_{l})), \end{array}$$

**ChemBERTa-v1** ChemBERTa-v1 is based on RoBERTa (Liu *et al.* 2019), a bidirectional transformer encoder model. ChemBERTa-v1 was trained on 77 million unique SMILES from PubChem (Kim *et al.* 2019) for 10 epochs, with a vocabulary size of 52K and a max sequence length of 512, using the masked language modeling (MLM) task; this task was inspired by Devlin et al. (Devlin *et al.* 2018). At training time, 15% of the tokens are replaced with a special [MASK] token, and the objective of the training is to predict what token was replaced.

**ChemBERTa-v2** Similar to ChemBERTa-v1, ChemBERTa-v2’s architecture is based on RoBERTa. It differs, however, in that the vocabulary size is 519 tokens which are based on the dictionary of the most common SMILES characters, and all SMILES are canonicalized.

There are two versions of ChemBERTa-v2, each use a different pretraining scheme. The first model uses the MLM pretraining task mentioned before, similar to ChemBERTa-v1. The other model uses a multi-task regression (MTR) objective, which is trained to simultaneously predict 200 different molecule properties for each molecule in the training dataset. These properties, such as ALOGP, PSA, and MW, can be calculated directly from the SMILES using RDKit (Landrum *et al.* 2006).

**T5Chem** The text-to-text transfer transformer (T5) (Raffel *et al.* 2020) models both classification and generation tasks in sequence-to-sequence fashion, i.e. taking text as input and producing new text as output, by using a transformer model consisting of both the transform encoder and transform decoder. Through self-supervised training, the model was pretrained on PubChem molecules and achieved state-of-the-art results on four distinct types of task-specific reaction prediction tasks.

### 2.4 Evaluation

In this section, we describe the datasets, evaluation metrics, and baselines used in our experiments.

**Datasets:** For future applications, the trained models must generalize well to other conditions and not overfit a single database source. Therefore, to approximate how our model will perform on real-world data, it is necessary to validate the models across databases. To accomplish this, in each evaluation, we use one database as the test set, while the rest of the databases are used for training. To prevent leakage of the test data into the training model, we remove duplicates from the training set; these are drugs that also appear in the test set with the same label. When handling our negative samples, i.e., drugs that were not withdrawn, we utilize DrugBank as our source of approved drugs.

When considering a given training or test database *D*, we consider an approved drug that was not withdrawn from database *D* a negative sample. In contrast, a drug withdrawn from database *D* constitutes a positive sample, the labeled samples are then used in two model evaluation schemes: the agreement split evaluation and the no agreement splits, each of which is based on cross-database evaluation. Table 1 presents the statistics when using the cross-database evaluation using each data splits. For the agreement split evaluation, drugs labeled as positive in the training set and negative in the test set, or vice versa are removed from the split completely. For the no agreement split evaluation, our assumption is that for a model to generalize well it should not just memorize the training set. Rather, it should understand the factors that contribute to a particular prediction. Based on this assumption, drugs whose label differs in the training and test sets are not removed.

**Table 1.** Number of drugs from each class in the training and test datasets for each of the database combinations used in our evaluation.

Training databases	Test database	Split type	#Train negative	#Train positive	#Test negative	#Test positive	#Test positive ratio
{DrugBank, NCATS, WITHDRAWN}	ChEMBL	No Agree	2676	1103	1949	616	0.316
{NCATS, WITHDRAWN, ChEMBL}	DrugBank	No Agree	2479	1519	2212	219	0.099
{DrugBank, WITHDRAWN, ChEMBL}	NCATS	No Agree	1757	597	2915	1098	0.3766
{DrugBank, NCATS, WITHDRAWN}	ChEMBL	Agree	2676	1103	1805	366	0.2027
{NCATS, WITHDRAWN, ChEMBL}	DrugBank	Agree	2479	1519	1816	166	0.0914
{DrugBank, WITHDRAWN, ChEMBL}	NCATS	Agree	1757	597	2773	828	0.2985

This creates inconsistencies in the data labeling, the data can be seen to mimic a real-world situation in which a drug has not been withdrawn but will be in the future.

**Metrics:** The main metric for assessing performance in our study is the area under the receiver operating characteristic curve (AUC) score. The receiver operating characteristic (ROC) curve is created by plotting the true positive rate (TPR) against the false positive rate (FPR) at various threshold settings. The true positive rate is also known as the recall or sensitivity. The false positive rate is also known as the fall-out. The AUC score is the area under the ROC curve and is equal to the probability that a classifier will rank a randomly chosen positive observation higher than a randomly chosen negative one. As part of our comprehensive evaluation, we also report the area under the precision–recall curve (PR-AUC). The precision–recall curve is calculated based on different precision and recall values at different cutoffs, and then the area under this curve is calculated. The PR-AUC is used to evaluate the performance of the prediction in the case of highly imbalanced data. Additionally, we report the accuracy, specificity, sensitivity and the Matthews correlation coefficient (MCC).

**Baselines:** Our evaluation involves benchmarking our models against several well-established ones. First, we replicate the model by Onay *et al.* (2017), using Linear, Medium, and Coarse Gaussian SVMs. Second, we adopt Yang et al.’s model (Yang *et al.* 2019) with a Directed Message Passing Neural Network for property prediction. Third, we use Jaeger et al.’s approach (Jaeger *et al.* 2018), feeding unsupervised feature representations for SMILES molecules into XGBoost (Chen and Guestrin 2016) and SVM (Cortes and Vapnik 1995). Fourth, we generate features using DeepChem (Ramsundar *et al.* 2019) and use XGBoost due to its handling of missing values. In the fifth and sixth models, we extract pre-trained embeddings from ChemBERTa-v1, Chemprop, Mordred, Mol2Vec, and ToxPrint and use these with various classifiers such as XGBoost, Random Forest, Logistic Regression, and SVM.

## 3 Results

This section provides results and findings analysis from our experiments.

### 3.1 Agreement split evaluation

Table 2, and Supplementary Tables S2 and S4 presents the results obtained in the agreement split evaluation. The best results are achieved by all models on the DrugBank and ChEMBL datasets, whereas the poorest results are obtained on the NCATS dataset. This can be attributed in part to the fact that DrugBank and ChEMBL are well-curated sources of drug information with a distribution that is easy for a model to learn.

**Table 2.** Drug withdrawal prediction results obtained in the agreement split evaluation.^a^

Test dataset	Input features	Model	AUC	PR-AUC	ACC	Sp	Sn	MCC
ChEMBL	SMILES	ChemBERTa v1	**0.8464**	**0.6503**	**0.8821**	0.9978	0.3115	**0.5107**
		ChemBERTa v2 MLM	0.644	0.2746	0.8314	**1**	0	0
		ChemBERTa v2 MTR	0.6423	0.2615	0.8314	**1**	0	0
		T5Chem	0.7783	**0.6000**	**0.8719**	0.9612	**0.4316**	**0.4798**
	Atom Descriptors	Chemprop	0.7029	0.3325	0.7927	0.8947	0.2896	0.2011
	Tox, Mordred	SVM	0.6033	0.228	0.8314	**1**	0	0
		XGBoost	0.6362	0.2642	0.7812	0.8964	0.2131	0.1252
	Mol2Vec	SVM	0.6371	0.2673	0.8314	**1**	0	0
		XGBoost	0.6348	0.2433	0.7789	0.8975	0.194	0.1062
	Tox, RDKit, ECFP, Mol2Vec, Mordred	XGBoost	0.6439	0.2606	0.7798	0.8925	0.224	0.131
	ChemBERTa, Chemprop, Mordred, ToxPrint, Mol2Vec		**0.7923**	0.5468	0.8623	0.9712	**0.3251**	0.4118
	ChemBERTa		0.7677	0.5082	0.8577	0.9717	0.2951	0.3834
DrugBank	SMILES	ChemBERTa v1	0.7237	0.2195	0.4859	0.4548	0.8253	0.1567
		ChemBERTa v2 MLM	0.6262	0.1748	0.8314	**1**	0	0
		ChemBERTa v2 MTR	0.6053	0.1184	**0.9062**	0.9879	0.012	0.0002
		T5Chem	**0.8047**	**0.3896**	0.3602	0.2824	**0.9216**	0.128
	Atom Descriptors	Chemprop	**0.7650**	**0.2315**	0.7194	0.7214	0.6988	**0.2508**
	Tox, Mordred	SVM	0.6498	0.1466	**0.9157**	**0.9994**	0	0.0068
		XGBoost	0.6740	0.1473	0.6635	0.6707	0.5843	0.1481
	Mol2Vec	SVM	0.6414	0.1291	0.8809	0.9548	0.0723	0.0354
		XGBoost	0.7046	0.1824	0.7230	0.7406	0.5301	0.1667
	Tox, RDKit, ECFP, Mol2Vec, Mordred	XGBoost	0.6817	0.1704	0.6524	0.6608	0.5602	0.1277
	ChemBERTa, Chemprop, Mordred, ToxPrint, Mol2Vec		0.7555	0.1996	0.5101	0.4769	0.8735	**0.1952**
	ChemBERTa		0.7464	0.1981	0.4975	0.4626	**0.8795**	0.1912
NCATS	SMILES	ChemBERTa v1	**0.6784**	**0.4018**	0.6906	0.7764	**0.4034**	0.1714
		ChemBERTa v2 MLM	0.5823	0.2767	**0.7701**	**1**	0	0
		ChemBERTa v2 MTR	0.5611	0.2737	**0.7701**	**1**	0	0
		T5Chem	**0.6884**	**0.4254**	0.6773	0.7147	**0.5519**	**0.2358**
	Atom Descriptors	Chemprop	0.5945	0.3513	0.7495	0.9073	0.2210	0.1649
	Tox, Mordred	SVM	0.5945	0.3513	**0.7701**	**1**	0	0
		XGBoost	0.5466	0.2630	0.7162	0.8835	0.1558	0.0499
	Mol2Vec	SVM	0.5805	0.2885	**0.7701**	**1**	0	0
		XGBoost	0.5703	0.2791	0.7287	0.9044	0.1401	0.0609
	Tox, RDKit, ECFP, Mol2Vec, Mordred	XGBoost	0.5735	0.2803	0.7331	0.9088	0.1449	0.0741
	ChemBERTa, Chemprop, Mordred, ToxPrint, Mol2Vec		0.6615	0.3837	0.7351	0.8615	0.3116	0.1903
	ChemBERTa		0.6643	0.3908	0.7345	0.8532	0.3370	**0.2038**

a The results presented are based on the agreement split in which all duplicates are removed. Bold text indicates the best and second best models for each performance metric.

Despite the use of the intermediate representation of SMILES introducing potential limitations in the data, and the recent popularity of graph-based input models over SMILES-based models Yang *et al.* (2019), our analysis of the performance of transformer models revealed otherwise. We found that pretrained SMILES-based models, such as T5Chem and ChemBERTa-v1, generalized effectively when tasked with predicting whether a drug was withdrawn from the market or not. These models demonstrated high performance across all datasets, achieving high AUC, MCC, Sensitivity and PR-AUC scores. Furthermore, the influence of incorporating pre-trained transformer embeddings becomes particularly noticeable when they are combined with supplementary features or employed solely with conventional machine learning models.

We believe that another potential advantage of using LLMs is the attention mechanism. This feature enables the model to understand contextual dependencies and focus on particular segments of the input sequence that are most relevant. As can be seen in Table 2, the predictive performance of Mol2Vec underperforms the performance of attention-based models or features. This can be explained by the fact that Mol2Vec is oblivious to the contextual relationship between different sections of the molecular graph. When different parts of the molecular graph are connected to other parts, they contribute differently. This allows the attention-based models to learn the connections between different parts of the graph, ultimately outperforming Mol2Vec.

ChemBERTa-v2 did not achieve the same performance as ChemBERTa-v1 and T5Chem. When viewing both the specificity and sensitivity metrics together, we receive the full picture which is the models fail to learn and only return the majority class as predictions. This is probably due to its limited vocabulary, which forces the model to utilize character-level representations for molecules; given this, ChemBERTa-v2 is unable to utilize common complex structures with larger vocabulary sizes when using WordPiece or SentencePiece tokenizers like those used by T5Chem and ChemBERTa-v1, and therefore it must rely only on atom-level representations.

The Chemprop model, based on a graph neural network (GNN), has proven to outperform the traditional methods, even achieving a higher PR-AUC than ChemBERTa-v1 on the DrugBank dataset. Nevertheless, it did not surpass the performance of transformer models or transformer embeddings. The reason behind this could be the unique information propagation scheme of the GNN, which, though similar to transformers in disseminating information between atoms and bonds, assigns equal weight to all data. Consequently, it does not effectively filter out irrelevant noise, which can compromise its prediction tasks. Therefore, the embeddings generated by transformers tend to be more effective in making predictions, either in the final transformer layer or when applied to classical models.

By comparing Supplementary Tables S2 and S4, it’s apparent that the application of ChemBERTa embeddings either leads to superior performance across most models and metrics, or the absolute difference in performance between the model using these embeddings and the one employing a variety of different embeddings and features together is notably small (<0.01). Furthermore, comparing the tables reveals a striking change in the SVM model’s behavior. Initially, constrained by its feature set, the SVM model could only predict zeros, leading to zero scores in both MCC and sensitivity (and achieve perfect score in specificity). However, the integration of ChemBERTa embeddings enabled the SVM model to effectively delineate a decision boundary. This marked improvement elevated its performance, making it comparable to that of other sophisticated learning algorithms.

### 3.2 No agreement split evaluation

Supplementary Tables S1, S3, and S5 outline the findings from the no agreement split evaluation. As detailed in Table 1, this split evaluation presents a higher positive ratio, a condition that ordinarily simplifies the learning process for supervised algorithms. Despite this, the inconsistent labeling encountered during the no agreement split evaluation posed a more complicated challenge.

Regardless, pretrained Language Representation Models (LLMs) or LLM embeddings, similar to the agreement split evaluation, demonstrated superior performance on metrics such as AUC, PR-AUC, ACC, and MCC. These models, excluding those that failed to converge and only predicted 0 s (which automatically yields a perfect score in specificity), secured the second best results in terms of specificity.

Mol2Vec’s performance was reduced in the no agreement split evaluation. This can be attributed to the nature of static embeddings, which have varying effectiveness across different tasks. For instance, without further fine-tuning, the embedding for the word “actor” may not perform well in fields such as cyber-security. This is because in most large general corpora, the term “actor” predominantly refers to a performer in the context of entertainment, not a cyber intruder or “hacker.” Thus, the limitations of context-specific embeddings become evident in diverse applications.

Moreover, we observed that traditional machine learning models, such as XGBoost, underperformed in the no agreement split evaluation. Specifically, XGBoost, when used with all hand-crafted features, yielded results inferior to random performance. The usage of ToxPrint and Mordred saw slightly improved results, but they were not considerably better than random performance. This underperformance might be attributed to XGBoost’s inherent limitation of depending on predefined features to predict drugs labeled differently in the training and test sets. Notably, traditional models witnessed improved performance when they were utilized either with a distinct set of embeddings or solely with ChemBERTa embeddings. This finding is consistent with patterns noted during the agreement split evaluation. As illustrated by a comparison of Supplementary Tables S3 and S4, it’s clear that the integration of ChemBERTa embeddings with all classifiers surpassed the combined use of various other embeddings and features on most metrics.

## 4 Discussion

In this research, we highlight the efficacy of pretrained Language Models (LLMs) in forecasting the potential withdrawal of drugs from the market.

The significance of this research lies in its ability to guide the development of an accurate predictive model, which relies solely on a drug’s molecular structure. This model can be utilized early in the drug development lifecycle, a phase often marked by limited information about the drug under study. Our model aids in preemptively identifying potential safety concerns, such as unexpected side effects, offering valuable insights before the initiation of the drug development process.

Our experiments indicated that pretrained LLMs, employing SMILES strings, outperformed other models despite earlier noted limitations. This superiority extended to when LLMs were used as embeddings for traditional ML models. The performance of our models was compared to that of graph-based models, static embedding models, and classical machine learning models with hand-crafted features.

Four databases were used to evaluate the models; in each round of our experiments a different database was selected as the test set in each instance, thus facilitating cross-database validation of our models. Moreover, we devised two unique splits for every evaluation stage. The first is a conventional split with strictly no overlap between instances within the training and test sets. In contrast, the second split permits label inconsistencies between the training and test sets, meaning drugs exhibiting different labels in these sets were intentionally preserved.

The no agreement split enabled us to analyze the performance in a simulated real-world setting. The subset of drugs in the test set with inconsistent labeling can be viewed as drugs currently on the market, which will, however, be labeled “withdrawn” in the future (which is how they are labeled in the test set). We examined our proposed models’ performance on this specific subset, which only contains drugs that are labeled as withdrawn in the test set but not in the training set, for each test dataset and compared our pretrained LLMs’ ability to identify withdrawn drugs to that of Chemprop, which served as a baseline. As shown in Table 3, when looking at those drugs that were labels as 0 in the training set but 1 in the test set (under the disagreement column), a pretrained LLM, such as ChemBERTa-v1 after fine-tuning for the task, outperformed Chemprop, achieving over 50% accuracy across all datasets. Accordingly, ChemBERTa-v1 could be used to identify the majority of drugs that will be withdrawn in the future due to discontinuation or adverse events.

**Table 3.** The number of drugs that are approved and not withdrawn in the training set (but are labeled as withdrawn in the test set), for which our model predicted, with high confidence, that the drug would be withdrawn from the market.

Model	Test dataset	Disagreement	Predicted	Accuracy
ChemBERTA-v1	DrugBank	42	29	**0.690**
	ChEMBL	220	111	**0.504**
	NCATS	234	131	**0.559**
Chemprop	DrugBank	42	17	0.404
	ChEMBL	220	71	0.322
	NCATS	234	70	0.299

Bold values indicates best performance.

In addition to performance analysis, we examined individual predictions of the drugs in this subset that were labeled as withdrawn in the test set. As ChEMBL’s website is highly informative about the reasons for a drug’s withdrawal (due to discontinuation or adverse events), we used the scenario in which the ChEMBL database was the test database for the no agreement split for this analysis. Based on the model’s prediction, we sorted all of the drugs and manually verified whether they were actually withdrawn from the market. Table 4 summarizes our findings; as can be seen, our ChemBERTA-v1 model was able to identify drugs that were withdrawn from the market due to ADRs with high confidence. For example, predicting the probability of drugs to be withdrawn on all our test drugs and sorting by their probability (as seen in the “Model Prediction” column in Table 4), we ranked our drugs from the most likely to be withdrawn to the least likely. Our model ranked Cianidanol as 4th, Novobiocin as 13th and Clometacin as 18th, and the first drug that is labeled as “not withdrawn” in our test set was ranked 158th. This demonstrates that our model was able to accurately identify potentially dangerous drugs and rank them higher than safe drugs. This is an important result, as it implies that our model can be used to identify potentially dangerous drugs before they are publicly available.

**Table 4.** Analysis of model prediction of withdrawn drugs that are labeled as “not withdrawn” in the training set and “withdrawn” in the test set.

Name	Description	Model prediction	Reason
Cianidanol	“An antioxidant flavonoid occurring especially in woody plants as both (+)-catechin and (-)-epicatechin (cis) forms.”	0.982	“Catechin and its metabolites can bind tightly to red blood cells and thereby induce the development of autoantibodies, resulting in haemolytic anaemia and renal failure. This resulted in the withdrawal of the catechin-containing drug Catergen, used to treat viral hepatitis, from the market in 1985.”
Novobiocin	“An antibiotic compound derived from Streptomyces niveus. It has a chemical structure similar to coumarin.”	0.967	“The oral form of the drug has since been withdrawn from the market due to a lack of efficacy.”
Clometacin		0.963	“Clometacin was withdrawn from the French market in 1990 because of concern regarding hepatotoxicity.”
Vincamine	“A monoterpenoid indole alkaloid obtained from the leaves of Vinca minor with a vasodilatory property.”	0.948	“It was withdrawn from the German market in 1987 for hematologic toxicity.”
Fenoterol	“An adrenergic beta-2 agonist that is used as a bronchodilator and tocolytic.”	0.939	“In the 1980s, concerns emerged about its safety and its use being associated with an increased risk of death. It was removed from the New Zealand drug tariff in 1989, because its introduction and widespread use was associated with an epidemic of asthma deaths.”
Fenclofenac	“A nonsteroidal anti-inflammatory drug (NSAID) previously used in rheumatism.”	0.930	“It has mild immunosuppressive effects and may displace thyroid hormone from its binding protein. It can also cause lichen planus. Due to its side effects, it was withdrawn from the UK and US in the 1980s.”
Rimonabant	“Rimonabant is an anorectic anti-obesity drug produced and marketed by Sanofi-Aventis. It is an inverse agonist for the cannabinoid receptor CB1. Its main avenue of effect is reduction in appetite.”	0.922	“It was first approved in Europe in 2006 but was withdrawn worldwide in 2008 due to serious psychiatric side effects; it was never approved in the US.”
Lumiracoxib	“A COX-2 selective non-steroidal anti-inflammatory drug (NSAID).”	0.903	“Lumiracoxib has been withdrawn from the market in several countries, mainly due to its potential for causing liver failure (sometimes requiring liver transplantation). It has never been approved for use in the US.”
Pentobarbital	“A barbiturate drug used to induce sleep, cause sedation, and control certain types of seizures.”	0.892	“Pentobarbital was widely abused and sometimes known as ”yellow jackets” due to the yellow capsule of the Nembutal brand. Pentobarbital in oral (pill) form is no longer commercially available.”
Diethylstilbestrol	“A synthetic nonsteroidal estrogen used in the treatment of menopausal and postmenopausal disorders.”	0.872	“The US National Cancer Institute recommends that children born to mothers who took DES undergo special medical exams on a regular basis to screen for complications as a result of the medication. Individuals who were exposed to DES during their mothers’ pregnancies are commonly referred to as “DES daughters” and “DES sons.” Since the discovery of the toxic effects of DES, it has largely been discontinued and is now is no longer marketed for the most part.”
Flosequinan	“It was approved in 1992 in the US and UK to treat people with heart failure who could not tolerate ACE inhibitors or digitalis.”	0.542	“A clinical trial called PROFILE was initiated to see if the drug could be useful in a wider population. The study was terminated early in 1993 due to increased mortality in the drug arm of the trial.”

A major limitation of our work is that deep learning models are not always explainable. The importance of explainability in medical research stems from the fact that such research is still very conservative in nature. This is because decisions based on medical research and the translation of medical research into clinical decisions and practice can have a profound impact on many people’s lives. Without the ability to provide a reason for a drug’s future withdrawal from the market, it will be difficult for drug developers and big pharma to adopt a model that can predict whether or not a drug will be discontinued. Another limitation is our approach to this problem as a binary classification problem. We adopted this approach, since it is difficult to determine with certainty why the drug was withdrawn from the market. Moreover, we do not distinguish between drugs that were withdrawn due to discontinuation and those that were withdrawn as a result of ADRs. It is possible that the distribution of a drug was stopped due to the release of an update to the drug itself or the discovery of a better molecule.

In our paper, we proposed the use of an LLM for the prediction of drug withdrawals. We also suggested that pretrained LLMs can assist in the drug development process by identifying (early on) candidate molecules with the greatest likelihood of being discontinued or resulting in an adverse response, as opposed to discovering these issues later in the drug development process after significant time and resources have been invested in its development or after a drug has been released, needlessly exposing patients to ADRs. By leveraging the insights gained by the attention mechanism regarding the relation between intra-substructures’ interactions, our model is able to determine whether or not a drug should be withdrawn from the market. As part of future research, we plan to expand our drug withdrawal datasets and improve our model’s ability to identify potential ADRs associated with a drug, either through multi-class prediction or explainability methods. This way, our model could provide more valuable and detailed insights, aiding the decision-making process in drug development.

## Supplementary Material

btad519_Supplementary_Data
