# Supplementary material for: Pretrained transformer models for predicting the withdrawal of drugs from the market
Source: Bioinformatics. 2023 Aug 23;39(8):btad519. doi: 10.1093/bioinformatics/btad519 (PMC10469107; doi:10.1093/bioinformatics/btad519)
Supplement: btad519_Supplementary_Data [file btad519_supplementary_data.pdf]

Pretrained Transformer Models for Predicting the Withdrawal of Drugs from the Market- Supplementary Information

Eyal Mazuz, Guy Shtar, Nir Kutsky, Lior Rokach, and Bracha Shapira

July 21, 2023

Test Dataset	Input Features	Model	AUC	PR-AUC	ACC	Sp	Sn	MCC
ChEMBL	SMILES	ChemBERTa v1	**0.8798**	**0.7667**	**0.8456**	0.9964	0.3685	**0.5414**
		ChemBERTa v2 MLM	0.6283	0.3168	0.7598	**1**	0	0
		ChemBERTa v2 MTR	0.5678	0.2879	0.8314	**1**	0	0
		T5Chem	**0.9238**	**0.8698**	**0.9068**	0.9620	**0.7321**	**0.7349**
	Atom Descriptors	Chemprop	0.6871	0.4265	0.7454	0.8846	0.3052	0.2205
	Tox, Mordred	SVM	0.5571	0.2733	0.7598	**1**	0	0
		XGBoost	0.5542	0.2671	0.6916	0.8630	0.1494	0.0152
	Mol2Vec	SVM	0.5952	0.3033	0.7598	**1**	0	0
		XGBoost	0.4679	0.2179	0.6710	0.8445	0.1218	0.0406
	Tox, RDKit, ECFP, Mol2Vec, Mordred		0.4732	0.225	0.6702	0.8374	0.1412	-0.0251
	ChemBERTa, Chemprop, Mordred, ToxPrint, Mol2Vec	XGBoost	0.8074	0.6558	0.8288	0.9561	**0.3977**	0.4735
	ChemBERTa		0.7964	0.6475	0.8277	0.9666	0.3880	0.4687
DrugBank	SMILES	ChemBERTa v1	0.6825	0.2029	0.4599	0.4263	**0.7991**	0.1314
		ChemBERTa v2 MLM	0.5981	**0.2059**	0.7137	0.7568	0.3790	0.0817
		ChemBERTa v2 MTR	0.5852	0.1170	0.8795	0.9638	0.0274	0.0136
		T5Chem	**0.9102**	**0.7685**	**0.9514**	**0.9679**	0.7853	**0.7190**
	Atom Descriptors	Chemprop	0.6908	0.1776	0.6503	0.6519	0.6347	0.1696
	Tox, Mordred	SVM	0.6034	0.1229	**0.9095**	**0.9995**	0.0000	0.0064
		XGBoost	0.5606	0.1013	0.5722	0.5782	0.5114	0.0518
	Mol2Vec	SVM	0.6011	0.1131	0.8626	0.9421	0.0594	0.0018
		XGBoost	0.5132	0.0893	0.5981	0.6157	0.4201	0.0211
	Tox, RDKit, ECFP, Mol2Vec, Mordred		0.5044	0.0855	0.54909	0.5497	0.4521	0.0010
	ChemBERTa, Chemprop, Mordred, ToxPrint, Mol2Vec	XGBoost	0.7087	0.1724	0.4829	0.4467	0.8493	**0.1717**
	ChemBERTa		**0.7148**	0.1898	0.4784	0.4412	**0.8539**	0.1715
NCATS	SMILES	ChemBERTa v1	**0.7080**	**0.5029**	0.6918	0.7979	**0.4098**	0.2115
		ChemBERTa v2 MLM	0.5848	0.3224	**0.7264**	**1**	0	0
		ChemBERTa v2 MTR	0.5188	0.2810	**0.7264**	**1**	0	0
		T5Chem	**0.7723**	**0.5477**	0.7057	0.7159	**0.6785**	**0.3602**
	Atom Descriptors	Chemprop	0.5974	0.4073	0.7164	0.8964	0.2386	0.1732
	Tox, Mordred	SVM	0.5174	0.2825	**0.7264**	**1**	0	0
		XGBoost	0.4958	0.2648	0.6504	0.8494	0.1220	0.0364
	Mol2Vec	SVM	0.5572	0.3202	**0.7264**	**1**	0	0
		XGBoost	0.4833	0.2579	0.6504	0.8494	0.1220	0.0364
	Tox, RDKit, ECFP, Mol2Vec, Mordred		0.4869	0.2581	0.6626	0.8696	0.1129	-0.0234
	ChemBERTa, Chemprop, Mordred, ToxPrint, Mol2Vec	XGBoost	0.6748	0.4642	0.7177	0.8563	0.3497	0.2295
	ChemBERTa		0.6812	0.4774	0.7199	0.8473	0.3816	**0.2483**

Table S1: Drug withdrawal prediction results obtained in the no agreement split evaluation. The results presented are based on the no agreement split in which drugs in the training set may appear in the test set with the opposite label. Bold text indicates the best and second best models for each performance metric.

Test Dataset	Model	AUC	PR-AUC	ACC	Sp	Sn	MCC
ChEMBL	XGBoost	**0.7923**	**0.5468**	**0.8623**	0.9712	0.3251	**0.4118**
	Random Forest	0.7817	0.4599	0.8471	0.9922	0.1311	0.2774
	Logistic Regression	0.7431	0.4730	0.7637	0.8111	**0.5301**	0.2964
	SVM	0.7083	0.4846	0.8314	**1**	0	0
DrugBank	XGBoost	0.7555	0.1996	0.5101	0.4769	0.8735	0.1952
	Random Forest	0.7737	0.2526	0.5373	0.5083	0.8554	**0.2017**
	Logistic Regression	**0.7582**	**0.2716**	0.2775	0.2197	**0.9096**	0.0811
	SVM	0.6891	0.1589	**0.9162**	**1**	0	0
NCATS	XGBoost	0.6615	**0.3837**	0.7351	0.8615	0.3116	0.1903
	Random Forest	0.6375	0.3793	**0.7753**	0.9719	0.1171	0.1742
	Logistic Regression	**0.6694**	0.3567	0.6504	0.6780	**0.5580**	**0.2094**
	SVM	0.5225	0.2807	0.7701	**1**	0	0

Table S2: Drug withdrawn prediction results. The results presented are based on the agreement split in which all duplicates are removed. Using ChemBERTa Chemprop, Mordred and Mol2Vec as input for classical machine learning algorithms.

Test Dataset	Model	AUC	PR-AUC	ACC	Sp	Sn	MCC
ChEMBL	XGBoost	**0.8074**	**0.6558**	**0.8288**	0.9651	0.3977	**0.4735**
	Random Forest	0.7832	0.5587	0.7891	0.9892	0.1558	0.2970
	Logistic Regression	0.7856	0.6247	0.7696	0.8245	**0.5958**	0.4015
	SVM	0.7688	0.6300	0.7598	**1**	0	0
DrugBank	XGBoost	0.7087	0.1724	0.4829	0.4467	0.8493	0.1717
	Random Forest	0.7155	0.2047	0.5138	0.4815	0.8402	**0.1850**
	Logistic Regression	**0.7212**	**0.2246**	0.2826	0.2224	**0.8904**	0.0790
	SVM	0.6961	0.1593	**0.9099**	**1**	0	0
NCATS	XGBoost	0.6748	**0.4642**	0.7177	0.8563	0.3497	**0.2295**
	Random Forest	0.6659	0.2356	**0.7348**	0.9540	0.1521	0.1796
	Logistic Regression	**0.6773**	0.4273	0.6509	0.6820	**0.5683**	0.2292
	SVM	0.5105	0.3278	0.7264	**1**	0	0

Table S3: Drug withdrawn prediction results. The results presented are based on the no agreement split in which drugs in the training set may appear in the test set with the opposite label. Using ChemBERTa Chemprop, Mordred and Mol2Vec as input for classical machine learning algorithms.

Test Dataset	Model	AUC	PR-AUC	ACC	Sp	Sn	MCC
ChEMBL	XGBoost	0.7677	0.5082	0.8577	0.9717	0.2951	**0.3834**
	Random Forest	**0.7976**	0.5215	0.8554	**0.9950**	0.1667	0.3427
	Logistic Regression	0.7000	0.4197	0.8116	0.8981	0.3852	0.2973
	SVM	0.7468	**0.5469**	**0.8752**	0.4703	**0.9945**	0.2869
DrugBank	XGBoost	0.7464	0.1981	0.4975	0.4626	0.8795	0.1912
	Random Forest	0.7426	0.2088	**0.5197**	0.4862	0.8855	0.2068
	Logistic Regression	0.7281	**0.2127**	0.4470	0.4097	0.8554	0.1507
	SVM	**0.7661**	0.1963	0.5151	**0.4976**	**0.9036**	**0.2135**
NCATS	XGBoost	**0.6643**	**0.3908**	0.7345	0.8532	0.3370	0.2038
	Random Forest	0.6493	0.3750	**0.7687**	**0.9564**	0.1401	0.1637
	Logistic Regression	0.6587	0.3485	0.6237	0.6260	**0.6159**	**0.2057**
	SVM	0.6566	0.3851	0.7434	0.8871	0.2621	0.1772

Table S4: Drug withdrawn prediction results. The results presented are based on the agreement split in which all duplicates are removed. Using only ChemBERTa embedding as input for classical machine learning algorithms.

Test Dataset	Model	AUC	PR-AUC	ACC	Sp	Sn	MCC
ChEMBL	XGBoost	0.7964	0.6475	0.8277	0.9666	0.3880	0.4687
	Random Forest	**0.8055**	0.6327	0.8062	**0.9944**	0.2110	0.3850
	Logistic Regression	0.7540	0.5870	0.7949	0.8974	**0.4708**	0.4001
	SVM	0.7971	**0.7044**	**0.8476**	0.9949	0.3815	**0.5470**
DrugBank	XGBoost	0.7148	0.1898	0.4784	0.4412	0.8539	0.1715
	Random Forest	0.7070	0.1767	0.4887	0.4539	0.8402	0.1702
	Logistic Regression	0.7072	**0.1996**	0.4311	0.3906	0.8402	0.1369
	SVM	**0.7310**	0.1817	**0.4965**	**0.4593**	**0.8721**	**0.1917**
NCATS	XGBoost	**0.6812**	0.4774	0.7199	0.8473	0.3816	**0.2483**
	Random Forest	0.6551	0.4449	**0.7371**	**0.9499**	0.1721	0.1967
	Logistic Regression	0.6734	0.4201	0.6290	0.6264	**0.6357**	0.2351
	SVM	0.6759	**0.4789**	0.7269	0.8827	0.3313	0.2321

Table S5: Drug withdrawn prediction results. The results presented are based on the no agreement split in which drugs in the training set may appear in the test set with the opposite label. Using only ChemBERTa embedding as input for classical machine learning algorithms.
